# Adiposity-based obesity classification and cardiometabolic and kidney outcomes: a longitudinal UK Biobank analysis

**DOI:** 10.1016/j.ebiom.2026.106272

**Published:** 2026-05-18

**Authors:** Sophie Gunnarsson, Cecilia Karlsson, Rashmi B. Prasad, Sara F. Hansson

**Affiliations:** a Department of Clinical Sciences, Diabetes and Endocrinology, CRC, Lund University, Malmö, Sweden; b Translational Science and Clinical Development, Research and Early Development, Cardiovascular, Renal and Metabolism (CVRM), BioPharmaceuticals R&D, AstraZeneca, Gothenburg, Sweden; c Late-stage Development, Cardiovascular, Renal and Metabolism (CVRM), BioPharmaceuticals R&D, AstraZeneca, Gothenburg, Sweden

**Keywords:** Obesity, Adiposity, Body fat percentage, Waist circumference, Anthropometric measurements, Cardiometabolic, Longitudinal outcome, MACE, T2D, CKD

## Abstract

**Background:**

Obesity is commonly diagnosed using BMI, which does not capture total adiposity or fat distribution, as highlighted by the recent Lancet D&E Commission report. We assessed the prognostic value of an adiposity-based classification combining body fat percentage (BF%) and waist circumference (WC) in relation to cardiometabolic and kidney outcomes and evaluated its concordance with BMI.

**Methods:**

We classified 489,311 UK Biobank participants into five risk groups (Groups 1–5) with increasingly adverse adiposity profiles based on BF%–WC. Associations with three-point major adverse cardiovascular events (3P-MACE), type 2 diabetes (T2D), and chronic kidney disease (CKD) were estimated using cumulative incidence and Cox PH models. Comparison with BMI categories was visualised with an alluvial plot.

**Findings:**

Over a median 13.1 years (IQR 1.7), 24,778 (5.1%) participants experienced 3P-MACE, 30,376 (6.2%) had incident T2D, and 14,906 (3.0%) had incident CKD. The BF%–WC classification yielded stepwise risk stratification across endpoints. Compared to the reference Group 1, Group 5 had significantly higher risk: age- and sex-adjusted HR 9.23 (95% CI 8.70–9.83) for T2D, 2.27 (2.10–2.41) for CKD, and 1.63 (1.60–1.71) for 3P-MACE. Discordance with BMI was notable: 32.6% of individuals in the high-risk Group 5 had a BMI in the normal-to-overweight range, while the overweight category spanned all BF%–WC risk groups.

**Interpretation:**

An adiposity-based classification integrating BF% and WC associates significantly with cardiometabolic and kidney outcomes and reveals discordance with BMI, reinforcing the Commission’s framing of obesity as a disease driven by excess adiposity.

**Funding:**

SciLifeLab & Wallenberg DDLS Program; Knut and Alice Wallenberg Foundation; Swedish Research Council (2021-02623); Vinnova (2023-04234).

**Research in context**

**Evidence before this study**

In January 2025, The Lancet Diabetes & Endocrinology Commission proposed a diagnostic framework for obesity that moves beyond BMI-only approaches. We searched PubMed for articles citing the Commission’s publication, limiting records to those published between January 14, 2025, and December 3, 2025, in any language and format. This search identified 344 records. Among original research, at least eight studies examined prevalence, primarily in US cohorts, and at least six assessed incident disease and mortality risks, comparing Commission-aligned definitions with BMI-defined obesity.

The evidence base, however, remains limited. Definitions of excess adiposity rely predominantly on waist circumference (WC) and WC-derived indices; endpoints are largely confined to cancer, cardiovascular events or mortality; and only two studies with limited sample sizes evaluated obesity prevalence using a combination of body fat percentage (BF%), to capture total adiposity, and WC, to approximate central adiposity, thereby reflecting both fat amount and distribution. Robust validation of such combined adiposity measures for broader cardiometabolic and kidney outcomes in large cohorts, and demonstration of incremental prognostic value over BMI, is therefore still lacking.

**Added value of this study**

This study provides long-term validation of an adiposity-based classification system that combines BF% and WC in a large, well-characterised European cohort (UK Biobank; N = 489,311), with a median follow-up of 13.1 years. It shows that integrating BF% and WC captures risk dimensions missed by BMI alone, directly supporting the Commission’s call for more comprehensive adiposity profiling. The classification yields graded risk stratification for major outcomes, including three-point major adverse cardiovascular events (3P-MACE), type 2 diabetes (T2D), and chronic kidney disease (CKD). The classification system enabled identification of a significant portion of individuals at high risk without BMI-defined obesity. For example, a group with normal-range BMI but an adverse adiposity profile had significantly higher risks: a 45% higher risk of 3P-MACE, a 58% higher risk of CKD, and over four times the risk of T2D compared to those with healthy adiposity profiles.

**Implications of all the available evidence**

Taken together, the evidence indicates that BMI alone is insufficient for accurate risk stratification, missing nearly one third of high-risk individuals who have a normal to overweight BMI. Therefore, integrating BF% and WC improves identification of high-risk adiposity phenotypes. These findings reinforce policy and guideline updates toward adiposity-based definitions of obesity and could inform treatment eligibility for anti-obesity therapies by targeting clinically relevant risk while reducing missed cases and overtreatment. Future research should optimise utility in diverse populations and age ranges, and evaluate implementation in real-world settings, including cost, feasibility, and equity considerations.

## Introduction

Obesity affects more than one billion people worldwide, and its prevalence has more than tripled since 1974.^1^ In clinical practice, diagnosis of obesity has most frequently relied on body mass index (BMI).^2^ Despite its accessibility as a screening tool, BMI alone is an imperfect marker for obesity-related risks. BMI lacks the capacity to distinguish adipose tissue–the biologically active driver of risk–from lean mass, nor does it capture fat distribution.^3^ Reliance on BMI alone can misclassify risk and impede appropriate diagnosis and management,^4^ given that individuals with normal BMI may also have elevated disease risks depending on their body composition.^5^, ^6^, ^7^, ^8^

To tackle this issue, The Lancet Diabetes & Endocrinology Commission proposed a diagnostic framework that redefines obesity as a condition characterised by excess adiposity, with or without abnormal distribution or function of adipose tissue.^9^ Aligned with other efforts that advocate incorporating adiposity-focused measurements into obesity diagnosis,^10^, ^11^, ^12^ this framework moves beyond a BMI-only approach by recommending confirmation of excess adiposity using measures such as waist circumference (WC) or direct fat measurements, to better capture risk-relevant phenotypes. Prior studies that have sought to validate the new framework have largely focused on using waist-derived indices (WC, waist-to-height, waist-to-hip ratio) to approximate central adiposity and have reported differences in prevalence and risk stratification when applying this new definition compared with BMI-defined obesity.^13^, ^14^, ^15^, ^16^ However, the incremental prognostic value of combining direct measures of fat amount with anthropometric indicators of central adiposity for incident disease risk remains uncertain.

To address this gap, we aimed to validate the prognostic performance of a classification system that combines body fat percentage (BF%) and WC,^17^ to more accurately represent adipose tissue quantity and distribution. Leveraging longitudinal electronic health records from the UK Biobank (UKB),^18^ we evaluated the BF%–WC classification system’s ability to identify individuals at elevated risk of obesity-related cardiometabolic and kidney outcomes. We then compared this approach with BMI categories to estimate the extent of classification discordance and characterised disease risk among individuals with discordant classifications.

## Methods

### Study design and population

The UKB is a large population-based cohort study conducted in the United Kingdom between 2006 and 2010, involving over 500,000 participants aged between 40 and 69 years at the time of recruitment.^18^ The baseline assessment involved a comprehensive collection of demographics and medical history data through touchscreen questionnaires and nurse-led interviews. The data collection process is described in more detail in the UKB protocol.^19^ Physical measurements and biological samples were also obtained at the time of recruitment.

The study complied with the Declaration of Helsinki and the Good Clinical Practice guidelines of the International Council for Harmonisation. All participants provided written informed consent for data collection and analysis and for long-term follow-up via linkage to electronic health records, including hospital admissions, death and cancer records. The Northwest Research Ethics Committee reviewed and approved the UKB’s study protocol and operational procedures (REC reference no. 06/MRE08/65). Access to data was granted under the UKB application ID 26041. Reporting follows the Strengthening the Reporting of Observational Studies in Epidemiology (STROBE) guideline.

### Cohort characteristics

Data from the baseline visit in the UKB were used for cross-sectional obesity classification. Anthropometric data including waist and hip circumference, weight, height, and BMI were extracted. Body composition was assessed using both bioelectrical impedance analysis (BIA) and dual energy X-ray absorptiometry (DXA) scan in the UKB. Bioimpedance data was chosen for the current analysis due to its availability for nearly all participants (97.7%). DXA-derived fat percentage was calculated as total fat mass divided by the sum of total fat mass and total fat-free mass. Age was determined by calculating the time from date of birth to the baseline assessment date. Ethnicity was self-reported and grouped into white, black or black British, Asian or Asian British, Chinese, mixed, and other ethnic group. Smoking status was categorised into non-smoker, current and former smoker using self-reported data. Socioeconomic status was evaluated using the Townsend deprivation index (TPI), where quintiles were computed and higher TPI indicates a higher level of socioeconomic deprivation.^20^ Alcohol consumption was assessed by self-reported drinking frequency on a six-level ordinal scale: (1) daily or almost daily, (2) 3–4 times per week, (3) 1–2 times per week, (4) 1–3 times per month, (5) special occasions only, and (6) never. Levels of physical activities were classified as low, moderate and high, based on metabolic equivalent of task (MET)-minutes per week, following the International Physical Activity Questionnaire.^21^

### BF%–WC classification system

**Fig. 1:**
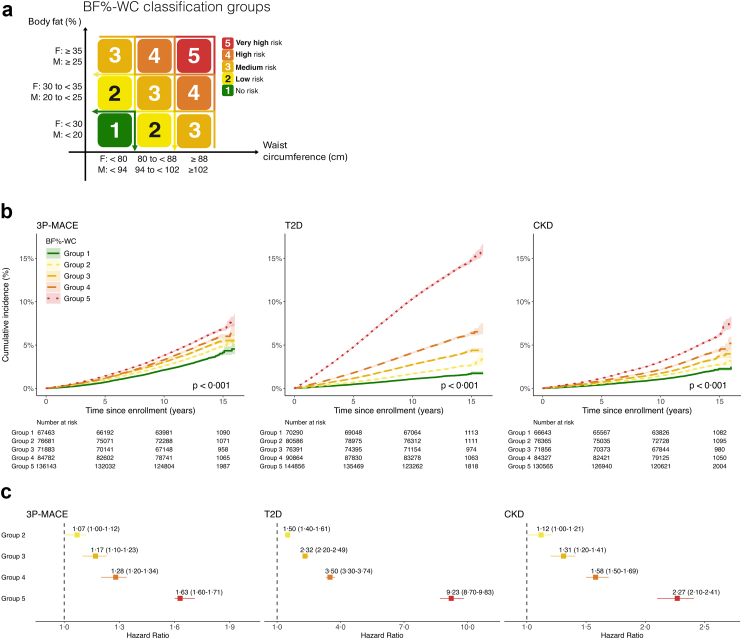
**Cardiometabolic and kidney outcomes in BF%–WC risk groups.** a) Classification matrix showing the 3 × 3 body fat percentage (BF%)-waist circumference (WC) risk stratification system using sex-specific cutoffs, resulting in five risk groups. b) Cumulative incidence curves for 3-point major adverse cardiovascular events (3P-MACE), type 2 diabetes (T2D), and chronic kidney disease (CKD), accounting for death as a competing risk. Shaded areas represent 95% confidence intervals. p-values were calculated using Gray’s test for competing risks. c) Age- and sex-adjusted hazard ratios (HRs) from Cox proportional hazards models for cardiometabolic and kidney outcomes across BF%–WC risk groups. Group 1 serves as the reference category (HR = 1.00). Error bars indicate 95% confidence intervals.

To apply the BF%–WC classification system to UKB participants, we excluded individuals with missing BMI, BF% or WC measurements, as well as those with BMI of <18.5 kg/m^2^. A complete-case analysis was performed given the low overall exclusion rate (2.3%, Supplementary Table S1). Participants were cross-classified into nine adiposity phenotypes using a 3 × 3 matrix of BF% and WC (Fig. 1a).^17^ Sex-specific thresholds defining elevated and high levels were based on WHO classifications and literature consensus cutoffs (BF%: 30% and 35% for females; 20% and 25% for males^22^, ^23^, ^24^; WC: 80 and 88 cm for females; 94 and 102 cm for males^25^). These nine phenotypes were subsequently aggregated into five risk groups following a traffic-light schema: Group 1 (no risk), Group 2 (slightly increased risk), Group 3 (increased risk), Group 4 (high risk), and Group 5 (very high risk).

### Cardiometabolic and kidney outcome definition

The endpoints assessed in this study were three-point major adverse cardiovascular events (3P-MACE), T2D and chronic kidney disease (CKD). To restrict analyses to incident disease, we excluded participants with pre-baseline T2D, cardiovascular disease, or kidney diseases, identified using ICD-10 codes (Supplementary Tables S2 and S3). For CKD, exclusions additionally incorporated baseline eGFR < 60 mL/min/1.73 m^2^ or UACR ≥ 30 mg/g. Follow-up time was calculated from baseline assessment to the earliest of: event occurrence, death or administrative censoring (2022-05-31), using the earliest censoring date across UKB data providers to ensure uniform data completeness for all participants. Cases inferred solely from self-report were excluded.

3P-MACE was defined as a composite outcome of nonfatal myocardial infarction (MI), nonfatal stroke, and cardiovascular death. Mortality outcomes were obtained from the death registries, provided by NHS England for participants in England & Wales and from the NHS Central Register (NHSCR) for participants in Scotland. Cardiovascular mortality was determined using ICD-10 codes ranging from I00 to I99, excluding codes for infection-related mortality, as previously described.^26^ Nonfatal MI and stroke were defined using a validated UKB algorithm integrating multiple data sources.^27^^,^^28^ Cases of T2D were derived from inpatient health records using ICD-10 code E11; outpatient data were unavailable at the time of analysis, which may have resulted in underestimation of incident T2D. Incident CKD cases were defined using ICD-10 code N18, which includes all five stages of CKD.^29^

### Statistical analyses

Pearson correlation was performed to test correlation between continuous variables. Correlation strength was interpreted using the absolute value of the Pearson coefficient (*r*), with moderate correlations defined as 0.5–0.7 and strong correlations defined as >0.7. Continuous risk factors were compared across obesity risk groups using the Kruskal–Wallis test; pairwise comparisons used the Wilcoxon rank-sum test. Categorical variables were compared using the chi-squared test.

To account for the competing risk of death, the cumulative incidence of cardiometabolic and kidney outcomes was visualised using the cumulative incidence function, and differences between BF%–WC risk groups using Gray’s test, calculated using the R package *tidycmprsk*.^30^ Associations between BF%–WC risk groups and time-to-event endpoints were estimated using cause-specific Cox proportional hazards models. Three sequential models were constructed with stepwise adjustment: Model 1 adjusted for age and sex; Model 2 additionally adjusted for BMI; and Model 3 further adjusted for lifestyle factors (physical activity, smoking status, alcohol consumption) and socioeconomic status (TPI quintiles). The proportional hazards assumption was evaluated using Schoenfeld residuals, where variables violating this assumption were stratified in final models. Potential effect modification was evaluated by testing multiplicative interactions between BF%–WC risk groups and age (<60 vs. ≥60 years), sex, smoking status, and physical activity level in fully adjusted Cox models using likelihood ratio tests. For variables with significant interactions (p < 0.05), stratified analyses were performed to estimate subgroup-specific associations.

All statistical tests were two-sided. p values from both descriptive analyses and Cox PH models were adjusted for multiple testing using the Bonferroni correction, with statistical significance defined as adjusted p < 0.05. All analyses and visualisations were performed in R version 4.1.0.

### Role of funders

The funders of the study did not have any role in study design, data collection, data analyses, data interpretation or writing of report.

## Results

### Cohort characteristics of the BF%–WC risk groups

Among the 500,641 UK Biobank participants, 489,311 (97.7%) had data on BIA-derived BF% and WC measurements. In the subset with both BIA- and DXA-derived BF% (N = 37,720), the measurements were highly correlated (Pearson’s *r* = 0.926; Supplementary Fig. S1), supporting the use of BIA-derived BF% for risk group classification.

After applying the BF%–WC classification to the UKB cohort, the distribution across the five risk groups is presented in Table 1. Consistent with previous findings,^17^ Group 5 (very high risk) was the most prevalent, comprising 32% (N = 155,769), followed by Group 4 (high risk; 19%, N = 95,102), Group 3 (increased risk; 16%, N = 79,947), Group 2 (slightly increased risk; 17%, N = 84,395), and Group 1 (no predicted risk; 15%, N = 74,098).

**Table 1:** Baseline characteristics of the BF%–WC risk groups in the UK Biobank.

Characteristics	BF%-WC risk groups					p-value^b^
	Group 1 N = 74,098 (15%)^a^	Group 2 N = 84,395 (17%)^a^	Group 3 N = 79,947 (16%)^a^	Group 4 N = 95,102 (19%)^a^	Group 5 N = 155,769 (32%)^a^	
Age (yr)	54 (47, 61)	56 (49, 62)	58 (50, 63)	59 (52, 64)	59 (52, 64)	<0.001
Sex						<0.001
Female	39,779 (54)	42,409 (50)	40,085 (50)	50,670 (53)	93,171 (60)	
Male	34,319 (46)	41,986 (50)	39,862 (50)	44,432 (47)	62,598 (40)	
Weight (kg)	63 (56, 72)	68 (62, 77)	73 (66, 82)	78 (70, 86)	90 (80, 100)	<0.001
BMI (kg/m^2^)	22.3 (21.0, 23.8)	24.3 (23.0, 25.6)	25.9 (24.5, 27.3)	27.6 (26.1, 29.1)	31.5 (29.2, 34.4)	<0.001
Body fat percentage (%)	21 (17, 27)	25 (22, 32)	30 (25, 35)	35 (28, 38)	39 (32, 44)	<0.001
Waist circumference (cm)	76 (70, 84)	81 (75, 89)	86 (79, 94)	88 (84, 98)	103 (94, 109)	<0.001
Hip circumference (cm)	95 (91, 98)	98 (95, 101)	101 (98, 104)	104 (100, 107)	110 (106, 116)	<0.001
WHR	0.81 (0.76, 0.87)	0.84 (0.77, 0.90)	0.87 (0.79, 0.93)	0.88 (0.81, 0.94)	0.92 (0.86, 0.98)	<0.001
Ethnicity group						<0.001
White	70,321 (95)	79,682 (95)	75,315 (95)	89,556 (95)	146,133 (94)	
Black	810 (1.1)	1097 (1.3)	1100 (1.4)	1457 (1.5)	3303 (2.1)	
Asian	859 (1.2)	1631 (1.9)	1817 (2.3)	2149 (2.3)	3075 (2.0)	
Others	654 (0.9)	717 (0.9)	688 (0.9)	848 (0.9)	1456 (0.9)	
Mixed	501 (0.7)	544 (0.6)	439 (0.6)	501 (0.5)	878 (0.6)	
Chinese	621 (0.8)	375 (0.4)	221 (0.3)	159 (0.2)	123 (<0.1)	

a Median (Q1, Q3); n (%).

b Kruskal-Wallis rank sum test; Pearson’s Chi-squared test.

Baseline characteristics by risk group are summarised in Table 1. Age increased modestly across groups, from a median of 54 years (IQR 14) in the reference Group 1 to 59 years (IQR 12) in Group 4, with a similar distribution in Group 5. While the distribution between sexes was more balanced in the lower-risk groups, the proportion of females increased from 54% in Group 1 to 60% in Group 5. All risk groups shared similar ethnic composition of the general UK population,^31^ with over 94% of participants self-reporting white ethnic origin across groups.

Anthropometric measures increased significantly across groups, including weight, BMI, BF%, WC, hip circumference and waist-to-hip ratio (WHR) (Kruskal–Wallis, all p < 0.001). Median BMI exceeded the overweight (≥25 kg/m^2^) threshold in Groups 3–5, and Group 5 reached the obesity (≥30 kg/m^2^) threshold (median BMI 31.5 kg/m^2^; IQR 5.2). In sex-stratified analyses (Supplementary Table S4), median WHR also exceeded high-risk thresholds in Groups 4–5 for females (≥0.85) and from Group 3 for males (≥0.90).^32^

### Prognostic values of the BF%–WC classification using longitudinal cardiometabolic and kidney outcomes

After excluding participants with reported events before the baseline assessment, the remaining cohort was followed for a median of 13.1 years (IQR 1.7). Between 2006 and 2022, a total of 24,778 (5.1%) participants experienced a 3P-MACE event, 30,376 (6.2%) participants developed T2D and 14,906 (3.0%) had incident CKD.

Cumulative incidence differed significantly across risk groups for all outcomes (Gray’s test, p < 0.001, Fig. 1b). At 15 years, Group 5 had the highest cumulative incidence (Supplementary Table S5): 3P-MACE 6.8% (95% CI 6.6–7.0), T2D 14.8% (14.5–15.1), CKD 6.2% (6.0–6.5), with event rates over twofold higher than the reference Group 1. Comparing Groups 5 and 4, the 15-year risk differences were 8.4 percentage point for T2D, 1.9 for CKD and 0.8 for 3P-MACE.

In age- and sex-adjusted Cox proportional hazards models (Model 1, Fig. 1c), higher BF%–WC risk groups were associated with progressively increased hazards across all endpoints. Using Group 1 as reference, adjusted HRs increased incrementally from 1.07 (1.00–1.12) in Group 2 to 1.17 (1.10–1.23) in Group 3, 1.28 (1.20–1.34) in Group 4, and 1.63 (1.60–1.71) in Group 5 for 3P-MACE. For T2D, the risk gradient was significantly more pronounced, with HRs of 1.50 (1.40–1.61), 2.32 (2.20–2.49), 3.50 (3.30–3.74), and 9.23 (8.70–9.83) for Groups 2 through 5, indicating a 2.64-fold increase from Group 4 to Group 5. For CKD, adjusted HR ranged from 1.12 (1.00–1.21) in Group 2 to 2.27 (2.10–2.41) in Group 5. The global likelihood-ratio test confirmed that the BF%–WC risk groups are significantly associated with increased risk for all endpoints (p < 0.001).

Additional adjustment for BMI attenuated effect sizes but preserved the graded associations (Model 2, Supplementary Table S6). For 3P-MACE, significant associations were observed only in the highest risk groups, with the adjusted HRs of 1.08 (1.03–1.14) in Group 4 and 1.19 (1.13–1.27) in Group 5. Similarly, associations with CKD were significant only in Groups 4 (1.14 [1.06–1.22]) and 5 (1.23 [1.14–1.33]). In contrast, associations with T2D remained robust across all groups, with HRs ranging from 1.24 (1.15–1.33) in Group 2 to 3.40 (3.17–3.64) for Group 5. This attenuation is consistent with substantial collinearity among adiposity measures (BMI–WC r = 0.81; BMI–BF% r = 0.57; Supplementary Fig. S2). Further adjustment of lifestyle and socioeconomic factors yielded comparable results (Model 3, Supplementary Table S6).

To investigate potential heterogeneity of the observed associations across different demographic subgroups, we conducted interaction testing. Modest effect modification by age and smoking status was observed for 3P-MACE, (interaction p = 0.023 and 0.012, respectively). For T2D, more significant interactions were observed for age (p < 0.001), sex (p < 0.001), and smoking status (p = 0.010). No interactions were detected for CKD and level of physical activity showed no effect modification across outcomes (p > 0.05). In stratified analyses, younger participants (<60 years) showed stronger associations with T2D than older participants (≥60 years), with adjusted HRs of 4.52 (4.01–5.10) and 2.89 (2.58–3.23) in Group 5, respectively (Supplementary Fig. S4). Sex-stratified analyses showed males had higher T2D risks in Groups 2–4, whereas females had slightly higher risk estimates in Group 5 (3.86 [3.36–4.43]) than males (3.28 [2.96–3.63]). For 3P-MACE, similar age-related patterns were observed, along with progressively diminishing sex differences from Group 2 through Group 5 (Supplementary Fig. S3). CKD showed no meaningful subgroup differences (Supplementary Fig. S5).

### Discordance between BF%–WC risk groups and BMI categories

**Fig. 2:**
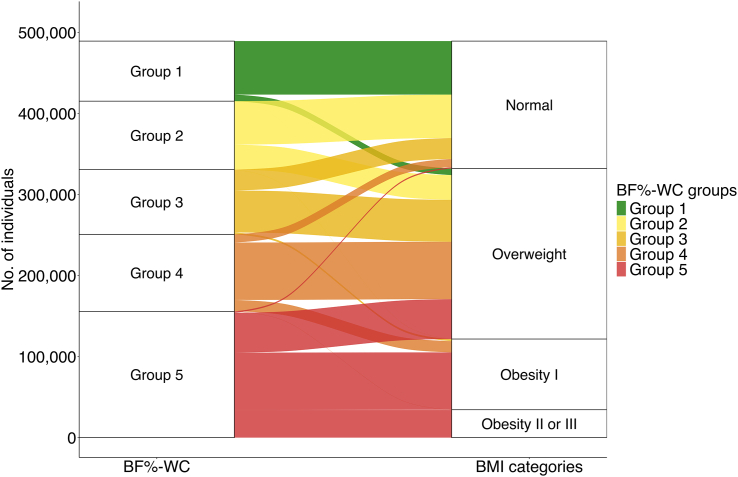
**Concordance between BF%–WC risk groups and BMI categories.** Alluvial plot illustrating participant distribution across BF%-WC risk groups and BMI categories (Normal weight – 18.5–24.9 kg/m^2^, Overweight – 25.0–29.9 kg/m^2^, Obesity I – 30.0–34.9 kg/m^2^, Obesity II – 35.0–39.9 kg/m^2^, Obesity III – ≥ 40.0 kg/m^2^). Flow width represents the proportion of individuals in each classification combination.

To illustrate how individual classifications differ across adiposity definitions, we constructed an alluvial plot mapping transitions from BF%–WC risk groups to BMI categories (Fig. 2). While there was broad concordance, important discordance was evident. Group 1 aligned predominantly with normal BMI (18.5–24.9 kg/m^2^), with 89.0% of its individuals falling within this range (Supplementary Table S7). Group 4 aligned largely with the overweight BMI category (25.0–29.9 kg/m^2^; 74.5%), whereas only 67.4% of Group 5 mapped to obesity classes (BMI ≥ 30.0 kg/m^2^), leaving nearly one-third of these very-high-risk individuals in the normal-to-overweight BMI range. The overweight BMI category, in turn, included individuals from all BF%–WC risk groups, with more than 35% of participants in each of Groups 2–4 classified as overweight, highlighting heterogeneity of adiposity profiles at this BMI level. Notably, nearly 5% of individuals in Groups 4–5, despite their substantial risk, were classified within the normal BMI range, suggesting potential risk of misclassification by BMI alone. These patterns mirror the residual risk carried by BF%–WC risk groups beyond BMI adjustment in Cox models, emphasising the added value of combined adiposity measures.

### Cardiometabolic and kidney risk in BMI-stratified BF%–WC risk groups

**Fig. 3:**
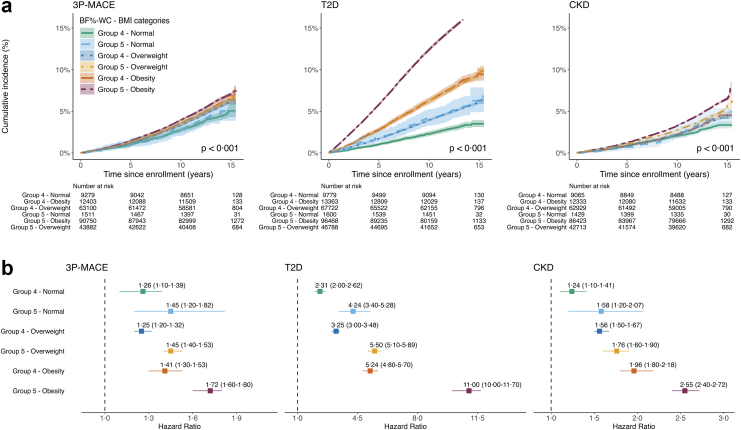
**Cardiometabolic and kidney outcomes in high-risk BF%–WC groups stratified by BMI categories.** a) Cumulative incidence curves for 3-point major adverse cardiovascular events (3P-MACE), type 2 diabetes (T2D), and chronic kidney disease (CKD) in BF%–WC risk groups 4–5 stratified by BMI categories (normal weight, overweight and obesity), accounting for death as a competing risk. Shaded areas represent 95% confidence intervals. p-values were calculated using Gray’s test for competing risks. b) Age- and sex-adjusted hazard ratios (HRs) from Cox proportional hazards models for cardiometabolic and kidney outcomes in BF%–WC risk groups 4–5 stratified by BMI categories (normal weight, overweight and obesity). Group 1 serves as the reference category (HR = 1.00). Error bars indicate 95% confidence intervals.

Given the high prevalence of individuals with BMI in the normal-to-overweight range among the high-risk adiposity groups (Groups 4 and 5), we quantified the clinical implications of this discordance by estimating the cumulative incidence and age- and sex-adjusted Cox proportional hazard ratios across Groups 4 and 5, stratified by BMI categories (normal, overweight, obesity). Among the high-risk strata, Group 4 with normal BMI had the lowest absolute risks. Consistent with the unstratified analysis, the concordant phenotype of Group 5 with obesity had the highest absolute risk across endpoints (Fig. 3a), with 15-year cumulative incidence of 7.0% (6.8–7.3) for 3P-MACE, 17.5% (17.1–17.8) for T2D and 6.5% (6.2–6.8) for CKD (Supplementary Table S8). Within each BMI category (normal, overweight, and obesity), Group 5 consistently had hazards at least as high as Group 4. For instance, using Group 1 as the reference, the age- and sex-adjusted HR for 3P-MACE in Group 5 with overweight BMI was 1.45 (95% CI 1.40–1.53; Fig. 3b), comparable with Group 4 with obesity (1.41 [1.30–1.53]). The discordant phenotype of Group 5 with normal-range BMI also showed significantly higher risks for 3P-MACE (1.45 [1.20–1.82]), T2D (4.24 [3.40–5.28]) and CKD (1.58 [1.20–2.07]). These results further highlight the added resolution for risk stratification by combining different adiposity measurements.

## Discussion

Although BMI remains widely used for obesity screening, recent evidence has increasingly recognised its limitations in assessing adiposity quantity, distribution, and metabolic function.^33^, ^34^, ^35^ Reflecting this paradigm shift, The Lancet Diabetes & Endocrinology Commission has reframed obesity as an adiposity-based disease and advocates moving beyond BMI-only approaches.^9^ Aligned with this framework, we adopted an adiposity-based classification system that captures two complementary dimensions of obesity-related risks: body fat percentage to quantify excess total adiposity, and waist circumference to capture abnormal (central) adiposity.^10^ Leveraging UK Biobank data, we validated this framework longitudinally over a median follow-up of 13.1 years: BF%–WC risk groups (Groups 1–5) showed clear prognostic stratification for 3P-MACE, T2D and CKD, with steepest gradient for T2D. Specifically, Group 5 with very high BF% and WC had substantially higher incidence risks compared to the reference Group 1, with over nine-fold higher risks for T2D, two-fold for CKD and a 64% higher risk for 3P-MACE, demonstrating the clinical utility of an adiposity-based classification system combining BF% and WC.

BF%–WC captures risk-relevant dimensions of adiposity that BMI incompletely reflects,^36^^,^^37^ as shown in two complementary analyses. First, while attenuated, associations for BF%–WC risk groups remained significant after adjustment for BMI, indicating additional risk not captured by BMI alone. The attenuation can largely be attributed to collinearity among adiposity measures, especially between BMI and waist circumference (Pearson’s *r* = 0.81). Second, within each BMI category (normal, overweight, obesity), Group 5 consistently had risks of incident disease higher than Group 4, underscoring added risk resolution beyond BMI. Since nearly one-third of Group 5 did not meet obesity thresholds by BMI, relying on BMI alone risks missing individuals with adverse adiposity profiles who are at materially elevated risk.

Heterogeneity within BMI categories helps explain these patterns. The overweight BMI category encompassed the full spectrum of BF%–WC risk groups, reflecting BMI’s limited ability to distinguish fat from muscle mass.^13^ Consistent with this observation, body fat percentage correlated only moderately with BMI (Pearson’s *r* = 0.57), indicating substantial variability in fat content at a given BMI, as previously reported.^38^ The strength of the association between body fat percentage and BMI has also been shown to differ across demographic strata, including age, sex and ethnicity.^39^ In particular, increasing age is associated with higher body fat percentage per BMI, plausibly reflecting age-related loss of lean mass coupled with increased fat mass in older adults.^40^

Subgroup analyses revealed stronger associations between BF%–WC risk groups and T2D among younger participants, consistent with prior research showing younger individuals developing T2D typically have higher BMI.^41^ This age-dependent pattern suggests that at older ages, non-adiposity factors may play a larger role in T2D pathogenesis, highlighting the potential value of early weight management intervention.

Notably, the BF%–WC risk groups showed strongest association with T2D, and this association persisted even after adjustment of BMI. This finding could be explained by the role of waist circumference as a proxy for visceral adiposity, a strong marker for metabolic dysfunction in obesity.^16^^,^^42^ While WC is an indirect measure of visceral adipose tissue,^43^ it remains a practical and cost-effective biomarker that consistently demonstrates robust correlation with imaging-based VAT quantification and with cardiometabolic risk markers.^44^, ^45^, ^46^ In this context, the WC component of the BF%–WC classification likely captures VAT expansion, which promotes ectopic fat deposition and the secretion of pro-inflammatory adipokines, thereby impairing insulin sensitivity.^47^ The resulting insulin resistance drives development of T2D and contributes to endothelial dysfunctional and vascular injury.^48^^,^^49^ These findings are consistent with the Cardiovascular-Kidney-Metabolic (CKM) framework,^50^ in which T2D is an early clinical manifestation of obesity-related metabolic derangements that precede downstream end-organ damage.^51^

Combining adiposity-based classification (BF%–WC) with BMI substantially improves identification of patients at risk. Within higher BF%–WC risk groups, BMI provided additional separation: Group 4 with obesity-range BMI had risks comparable to Group 5 with overweight BMI, and Group 5 with obesity-range BMI had the highest risk. These findings suggest that, while BMI should not be used in isolation, it can provide complementary information when incorporated into a more sophisticated, multi-metric framework, consistent with current recommendations for comprehensive adiposity assessment.^35^^,^^52^, ^53^, ^54^

Our findings have several practical implications. Incorporating BF% and WC alongside BMI can sharpen risk stratification, identify high-risk individuals who would be overlooked by BMI screening alone, and inform prioritisation for therapy. For event-driven outcome trials, using BF%–WC in addition to BMI could enrich for events and improve efficiency. In clinical practice, BF%, WC, and BMI should function as complementary screening flags that prompt further metabolic evaluation (e.g., glycaemia, lipids) rather than rigid composite gate for treatment eligibility.

This study has several limitations that should be considered in interpreting these findings. First, baseline blood samples were non-fasting,^55^ precluding assessment of insulin resistance status using fasting glucose and insulin. Second, recruitment was restricted to adults aged 40–69 years, limiting generalisability of our findings to a wider age group. Third, non-white ethnic groups were under-represented in UKB, broadly mirroring the general UK population,^31^ which limited our power to examine ethnic differences in obesity-related outcomes. Fourth, incident disease was ascertained based on ICD-10 codes from inpatient records only, which likely underestimated T2D diagnosis and would attenuate hazard ratios toward the null. Fifth, from a methodological perspective, we applied uniform sex-specific thresholds for BF% and WC without adjustment for ethnicity. Although ethnic-specific thresholds have been proposed for cardiometabolic risk stratification,^56^, ^57^, ^58^ the potential for misclassification in our analysis was likely limited due to the predominantly white ethnic composition of the cohort (>94%). Sixth, whilst excluded individuals had significantly lower BF% and BMI, this primarily reflects the study’s exclusion of participants with BMI < 18.5 kg/m^2^, rather than systematic differences. The similarity between included participants and the overall cohort suggests minimal selection bias. Finally, BF% was estimated using BIA due to data availability. While BIA correlates strongly with DXA-derived measures, studies have highlighted that it systematically underestimates BF% by approximately 3%,^59^, ^60^, ^61^ which may have led to underestimation of the proportion of participants classified into high-risk BF%-WC groups.

In conclusion, the BF%–WC classification provides robust, stepwise risk stratification for cardiometabolic and kidney outcomes and clear clinical utility, evidenced by consistent associations with longitudinal endpoints. These results underscore the value of refined obesity classification for improving risk stratification and guiding targeted prevention and treatment. Ultimately, this work provides robust, longitudinal evidence that adopting a comprehensive, adiposity-focused diagnostic framework represents a clinical necessity for accurately identifying and managing obesity-related disease risk.

## Contributors

SG, CK, RBP, and SFH contributed to the conception of the work. SG and SFH contributed to the data analysis. SG and CK drafted the article. All authors contributed to the interpretation of data and revision of the article. All authors gave final approval of the version to be published. SG and SFH had full access to raw data and were responsible for the decision to submit for publication.

## Data sharing statement

The data supporting the conclusions of this study are available from the UK Biobank Resource. Information on registration for data access can be found at http://www.ukbiobank.ac.uk/register-apply/. Data for this study were obtained under Resource Application Number 26041.

## Declaration of generative AI and AI-assisted technologies in the manuscript preparation process

During the preparation of this work the authors used the AstraZeneca Claude 4.5 Sonnet for improving language clarity. The authors have reviewed and confirmed the validity of the text and take full responsibility for the content of the published article.

## Declaration of interests

SG, CK, and SFH are AstraZeneca employees. SG, CK, RBP, and SFH hold stocks in AstraZeneca. RBP also holds stocks in Novo Nordisk.
