# Supplementary material for: Adiposity-based obesity classification and cardiometabolic and kidney outcomes: a longitudinal UK Biobank analysis
Source: eBioMedicine. 2026 May 18;128:106272. doi: 10.1016/j.ebiom.2026.106272 (PMC13261713; doi:10.1016/j.ebiom.2026.106272)
Supplement: Supplementary Figs. S1–S5 and Tables S1–S8 [file mmc1.pdf]

Supplementary Information

Adiposity-based obesity classification and cardiometabolic and kidney outcomes: a longitudinal UK biobank analysis

Authors:

Sophie Gunnarsson[1,3*], Cecilia Karlsson[2], Rashmi B. Prasad[3‡], Sara F. Hansson[1‡]

[1] Translational Science and Clinical Development, Research and Early Development, Cardiovascular, Renal and Metabolism (CVRM), BioPharmaceuticals R&D, AstraZeneca, Gothenburg, Sweden

[2] Late-stage Development, Cardiovascular, Renal and Metabolism (CVRM), BioPharmaceuticals R&D, AstraZeneca, Gothenburg, Sweden

[3] Department of Clinical Sciences, Diabetes and Endocrinology, CRC, Lund University, Malmö, Sweden

‡ These authors jointly directed this study

*Corresponding author: Sophie Gunnarsson, sophie·gunnarsson@med.lu.se

Table S1. Baseline characteristics of total cohort, included study participants, and excluded individuals due to BMI < 18·5 kg/m² or missing measurements for BMI, body fat percentage (BF%), or waist circumference (WC). NA, data not available.

**Characteristic**	**Excluded** N = 11,330 (2·3%)[1]	**Included** N = 489,311 (97·7%)[1]	**Total** N = 500,641 (100%)[1]
**Age (yr)**	59 (51, 64)	58 (50, 63)	58 (50, 63)
**Sex**			
Female	6,347 (56)	266,114 (54)	272,461 (54)
Male	4,983 (44)	223,197 (46)	228,180 (46)
**Weight (kg)**	50 (46, 55)	77 (67, 88)	76 (67, 88)
NA	8,535 (75)	2 (0·0004)	8,537 (1·7)
**BMI (kg/m²)**	18·0 (17·5, 18·4)	26·8 (24·2, 29·9)	26·7 (24·1, 29·9)
NA	8,417 (74)	0 (0)	8,417 (1·7)
**Body fat percentage (%)**	20 (15, 23)	31 (25, 38)	31 (25, 38)
NA	8,816 (78)	0 (0)	8,816 (1·8)
**Waist circumference (cm)**	87 (73, 99)	90 (81, 99)	90 (80, 99)
NA	445 (3·9)	0 (0)	445 (0·09)
**Hip circumference (cm)**	100 (91, 107)	102 (97, 108)	102 (97, 108)
NA	475 (4·2)	29 (0·0059)	504 (0·1)
**WHR**	0·86 (0·78, 0·94)	0·87 (0·80, 0·94)	0·87 (0·80, 0·94)
NA	521 (4·6)	29 (0·0059)	550 (0·1)
**Ethnicity group**			
White	10,448 (94)	461,007 (95)	471,455 (95)
Black	171 (1·5)	7,767 (1·6)	7,938 (1·6)
Asian	218 (2·0)	9,531 (2·0)	9,749 (2·0)
Others	125 (1·1)	4,363 (0·9)	4,488 (0·9)
Mixed	76 (0·7)	2,863 (0·6)	2,939 (0·6)
Chinese	57 (0·5)	1,499 (0·3)	1,556 (0·3)
NA	235 (2·1)	2,281 (0·5)	2,516 (0·5)

[1] Median (Q1, Q3); n (%)

Table S2. ICD codes for excluding participants with history of cardiovascular diseases before baseline

ICD codes	Disease description
**I11**	Hypertensive heart disease
**I13**	Hypertensive heart and chronic kidney disease
**I20**	Angina pectoris
**I21**	Acute myocardial infarction
**I22**	Subsequent ST elevation (STEMI) and non-ST elevation (NSTEMI) myocardial infarction
**I23**	Certain current complications following ST elevation (STEMI) and non-ST elevation (NSTEMI) myocardial infarction (within the 28-day period)
**I24**	Other acute ischemic heart diseases
**I25**	Chronic ischemic heart disease
**I34**	Nonrheumatic mitral valve disorders
**I35**	Nonrheumatic aortic valve disorders
**I37**	Nonrheumatic pulmonary valve disorders
**I42**	Cardiomyopathy
**I44**	Atrioventricular and left bundle-branch block
**I46**	Cardiac arrest
**I50**	Heart failure
**I51**	Complications and ill-defined descriptions of heart disease
**I62**	Other and unspecified nontraumatic intracranial hemorrhage
**I63**	Cerebral infarction
**I65**	Occlusion and stenosis of precerebral arteries, not resulting in cerebral infarction
**I66**	Occlusion and stenosis of cerebral arteries, not resulting in cerebral infarction
**I67**	Other cerebrovascular diseases

Table S3. ICD codes for excluding participants with history of kidney diseases before baseline

ICD codes	Disease description
**I12**	Hypertensive chronic kidney disease
**I13**	Hypertensive heart and chronic kidney disease
**N00**	Acute nephritic syndrome
**N01**	Rapidly progressive nephritic syndrome
**N02**	Recurrent and persistent hematuria
**N03**	Chronic nephritic syndrome
**N04**	Nephrotic syndrome
**N05**	Unspecified nephritic syndrome
**N06**	Isolated proteinuria with specified morphological lesion
**N07**	Hereditary nephropathy
**N08**	Glomerular disorders in diseases classified elsewhere
**N10**	Acute pyelonephritis
**N11**	Chronic tubulo-interstitial nephritis
**N12**	Tubulo-interstitial nephritis
**N13**	Obstructive and reflux uropathy
**N15**	Other renal tubulo-interstitial diseases
**N16**	Renal tubulo-interstitial disorders in diseases classified elsewhere
**N17**	Acute kidney failure
**N18**	Chronic kidney disease (CKD)
**N19**	Unspecified kidney failure
**N25**	Disorders resulting from impaired renal tubular function
**N26**	Unspecified contracted kidney
**N27**	Small kidney of unknown cause
**Q60**	Renal agenesis and other reduction defects of kidney
**Q61**	Cystic kidney disease
**Q62**	Congenital obstructive defects of renal pelvis and congenital malformations of ureter
**Q63**	Other congenital malformations of kidney
**R34**	Anuria and oliguria
**Z49**	Encounter for care involving renal dialysis

Table S4. Sex-stratified baseline characteristics of BF%-WC risk groups in the UK Biobank

**Female**					
	**Group 1** N = 39779 (15%)[1]	**Group 2** N = 42409 (16%)[1]	**Group 3** N = 40085 (15%)[1]	**Group 4** N = 50670 (19%)[1]	**Group 5** N = 93171 (35%)[1]
Age (yr)	53 (46, 60)	56 (49, 62)	58 (50, 63)	59 (51, 64)	59 (52, 64)
Weight (kg)	57 (53, 61)	62 (58, 66)	66 (62, 70)	71 (67, 75)	82 (76, 91)
BMI (kg/m²)	21·6 (20·5, 22·7)	23·4 (22·4, 24·5)	25·0 (23·9, 26·3)	26·9 (25·5, 28·4)	31·2 (28·8, 34·5)
Body fat percentage (%)	27 (24, 29)	32 (31, 34)	35 (34, 37)	38 (36, 40)	43 (40, 46)
Waist circumference (cm)	71 (68, 74)	75 (72, 77)	79 (76, 82)	84 (82, 86)	96 (91, 103)
Hip circumference (cm)	93 (90, 96)	97 (94, 100)	100 (97, 103)	103 (100, 106)	111 (106, 118)
WHR	0·76 (0·73, 0·79)	0·77 (0·74, 0·80)	0·79 (0·75, 0·83)	0·81 (0·78, 0·85)	0·87 (0·83, 0·91)
Ethnicity group					
Asian	360 (0·9)	544 (1·3)	681 (1·7)	992 (2·0)	1,845 (2·0)
Black	300 (0·8)	375 (0·9)	444 (1·1)	792 (1·6)	2,557 (2·8)
Chinese	410 (1·0)	195 (0·5)	133 (0·3)	102 (0·2)	92 (<0·1)
Mixed	299 (0·8)	300 (0·7)	275 (0·7)	301 (0·6)	617 (0·7)
Others	386 (1·0)	323 (0·8)	324 (0·8)	454 (0·9)	992 (1·1)
White	37,896 (96)	40,541 (96)	38,086 (95)	47,824 (95)	86,634 (93)

**Male**					
	**Group 1** N = 34319 (15%)[1]	**Group 2** N = 41986 (19%)[1]	**Group 3** N = 39862 (18%)[1]	**Group 4** N = 44432 (20%)[1]	**Group 5** N = 62598 (28%)[1]
Age (yr)	54 (47, 61)	57 (49, 63)	58 (50, 64)	59 (52, 64)	60 (52, 64)
Weight (kg)	73 (67, 78)	77 (72, 82)	82 (77, 88)	87 (82, 92)	99 (92, 108)
BMI (kg/m²)	23·3 (22·0, 24·7)	25·2 (24·0, 26·4)	26·7 (25·5, 28·0)	28·3 (27·0, 29·6)	31·8 (29·9, 34·3)
Body fat percentage (%)	17·1 (14·8, 18·7)	22·1 (20·9, 23·5)	24·5 (22·9, 26·3)	27·2 (25·8, 29·1)	30·8 (28·5, 33·5)
Waist circumference (cm)	84 (80, 88)	89 (86, 92)	94 (91, 97)	98 (96, 100)	108 (105, 114)
Hip circumference (cm)	97 (94, 100)	99 (96, 102)	102 (99, 105)	104 (101, 107)	110 (106, 114)
WHR	0·87 (0·84, 0·90)	0·90 (0·87, 0·92)	0·92 (0·90, 0·95)	0·94 (0·92, 0·97)	0·99 (0·96, 1·03)
Ethnicity group					
Asian	499 (1·5)	1,087 (2·6)	1,136 (2·9)	1,157 (2·6)	1,230 (2·0)
Black	510 (1·5)	722 (1·7)	656 (1·7)	665 (1·5)	746 (1·2)
Chinese	211 (0·6)	180 (0·4)	88 (0·2)	57 (0·1)	31 (<0·1)
Mixed	202 (0·6)	244 (0·6)	164 (0·4)	200 (0·5)	261 (0·4)
Others	268 (0·8)	394 (0·9)	364 (0·9)	394 (0·9)	464 (0·7)
White	32,425 (95)	39,141 (94)	37,229 (94)	41,732 (94)	59,499 (96)

[1] Median (Q1, Q3); n (%)

Table S5. Cumulative incidence of cardiometabolic and kidney outcomes in BF%-WC risk groups. Five-year and 15-year cumulative incidence (%) 95% confidence intervals (CIs) for 3P-MACE, T2D and CKD in BF%-WC risk groups, accounting for death as a competing risk.

**BF%-WC risk groups**		
**3P-MACE**	Years 5 (95% CI)	Years 15 (95% CI)
Group 1	0·7% (0·7% - 0·8%)	4·2% (3·9% - 4·5%)
Group 2	1·0% (0·9% - 1·0%)	4·9% (4·6% - 5·2%)
Group 3	1·2% (1·1% - 1·2%)	5·5% (5·3% - 5·8%)
Group 4	1·2% (1·1% - 1·2%)	6·0% (5·8% - 6·2%)
Group 5	1·4% (1·4% - 1·5%)	6·8% (6·6% - 7·0%)
**T2D**		
Group 1	0·5% (0·4% - 0·5%)	1·7% (1·5% - 2·0%)
Group 2	0·7% (0·7% - 0·8%)	2·7% (2·5% - 2·9%)
Group 3	1·2% (1·1% - 1·2%)	4·4% (4·1% - 4·7%)
Group 4	1·8% (1·7% - 1·9%)	6·4% (6·1% - 6·6%)
Group 5	4·8% (4·7% - 4·9%)	14·8% (14·5% - 15·1%)
**CKD**		
Group 1	0·4% (0·4% - 0·5%)	2·2% (2·0% - 2·5%)
Group 2	0·6% (0·5% - 0·6%)	2·7% (2·6% - 2·9%)
Group 3	0·7% (0·7% - 0·8%)	3·7% (3·4% - 4·0%)
Group 4	0·9% (0·8% - 0·9%)	4·3% (4·2% - 4·5%)
Group 5	1·2% (1·1% - 1·2%)	6·2% (6·0% - 6·5%)

Table S6. Multivariable Cox proportional hazards model for BF%-WC risk groups and incident cardiometabolic and kidney outcomes. Hazard ratios (HRs) with 95% confidence intervals (CIs) for 3P-MACE, T2D and CKD. Model 2: adjusted for age, sex, and BMI. Model 3: Model 2 plus physical activity, smoking status, alcohol consumption, and Townsend deprivation index quintiles.

	**3P-MACE**		**T2D**		**CKD**	
	HR (95% CI)	P-value	HR (95% CI)	P-value	HR (95% CI)	P-value
**Model 2**						
Group 2	1·00 (0·95 - 1·06)	1	1·24 (1·15 - 1·33)	1·31e-07	1·00 (0·93 - 1·08)	1
Group 3	1·04 (0·99 - 1·10)	0·36	1·63 (1·52 - 1·75)	< 2e-16	1·05 (0·98 - 1·13)	0·51
Group 4	1·08 (1·03 - 1·14)	0·011	2·07 (1·93 - 2·22)	< 2e-16	1·14 (1·06 - 1·22)	1·42e-03
Group 5	1·19 (1·13 - 1·27)	1·03e-08	3·40 (3·17 - 3·64)	< 2e-16	1·23 (1·14 - 1·33)	8·37e-07
**Model 3**						
Group 2	1·04 (0·98 - 1·10)	0·73	1·31 (1·20 - 1·43)	1·04e-08	1·01 (0·93 - 1·10)	1
Group 3	1·08 (1·02 - 1·15)	0·022	1·73 (1·59 - 1·88)	< 2e-16	1·09 (1·00 - 1·18)	0·16
Group 4	1·10 (1·04 - 1·17)	0·0043	2·20 (2·03 - 2·38)	< 2e-16	1·14 (1·05 - 1·24)	0·0052
Group 5	1·18 (1·10 - 1·26)	7·59e-06	3·54 (3·26 - 3·84)	< 2e-16	1·22 (1·12 - 1·34)	5·04e-05

Table S7. BMI Category Distribution by BF%-WC risk groups (N, %)

	BMI categories				
**BF%-WC groups**	Normal	Overweight	Obesity I	Obesity II	Obesity III
Group 1	65,971 (89·0)	8,115 (10·9)	12 (0·016)	0 (0·0)	0 (0·0)
Group 2	53,608 (63·5)	30,530 (36·2)	256 (0·30)	1 (0·001)	0 (0·0)
Group 3	25,837 (32·3)	51,771 (64·8)	2,315 (2·9)	20 (0·025)	4 (0·005)
Group 4	10,133 (10·7)	70,822 (74·5)	13,912 (14·6)	228 (0·24)	7 (0·0074)
Group 5	1,678 (1·1)	49,167 (31·6)	70,766 (45·4)	24,624 (15·8)	9,534 (6·1)

Table S8. Cumulative incidence of cardiometabolic and kidney outcomes in BF%-WC risk groups 4-5 stratified by BMI category. Five-year and 15-year cumulative incidence (%) 95% confidence intervals (CIs) for 3P-MACE, T2D and CKD in BF%-WC risk groups, accounting for death as a competing risk.

**BF%-WC risk groups by BMI categories**		
**3P-MACE**	Years 5 (95% CI)	Years 15 (95% CI)
Group 4 - Normal	1·0% (0·8% - 1·2%)	5·0% (4·3% - 5·8%)
Group 5 - Normal	1·1% (0·6% - 1·7%)	5·1% (3·9% - 6·5%)
Group 4 - Overweight	1·2% (1·1% - 1·3%)	6·1% (5·8% - 6·3%)
Group 5 - Overweight	1·3% (1·2% - 1·4%)	6·5% (6·1% - 6·9%)
Group 4 - Obesity	1·3% (1·1% - 1·5%)	6·4% (5·9% - 7·0%)
Group 5 - Obesity	1·5% (1·4% - 1·6%)	7·0% (6·8% - 7·3%)
**T2D**		
Group 4 - Normal	1·0% (0·8% - 1·2%)	3·5% (3·1% - 3·9%)
Group 5 - Normal	1·9% (1·3% - 2·7%)	6·3% (4·9% - 7·9%)
Group 4 - Overweight	1·7% (1·6% - 1·8%)	6·2% (5·9% - 6·5%)
Group 5 - Overweight	2·7% (2·6% - 2·9%)	9·6% (9·2% - 10·1%)
Group 4 - Obesity	2·8% (2·5% - 3·1%)	9·4% (8·7% - 10·2%)
Group 5 - Obesity	5·8% (5·7% - 5·9%)	17·5% (17·1% - 17·8%)
**CKD**		
Group 4 - Normal	0·8% (0·6% - 1·0%)	3·3% (2·9% - 3·8%)
Group 5 - Normal	0·7% (0·4% - 1·3%)	4·2% (3·2% - 5·4%)
Group 4 - Overweight	0·9% (0·8% - 0·9%)	4·4% (4·2% - 4·7%)
Group 5 - Overweight	1·1% (1·0% - 1·2%)	5·6% (5·2% - 6·1%)
Group 4 - Obesity	0·8% (0·7% - 1·0%)	4·5% (4·1% - 5·0%)
Group 5 - Obesity	1·2% (1·1% - 1·3%)	6·5% (6·2% - 6·8%)

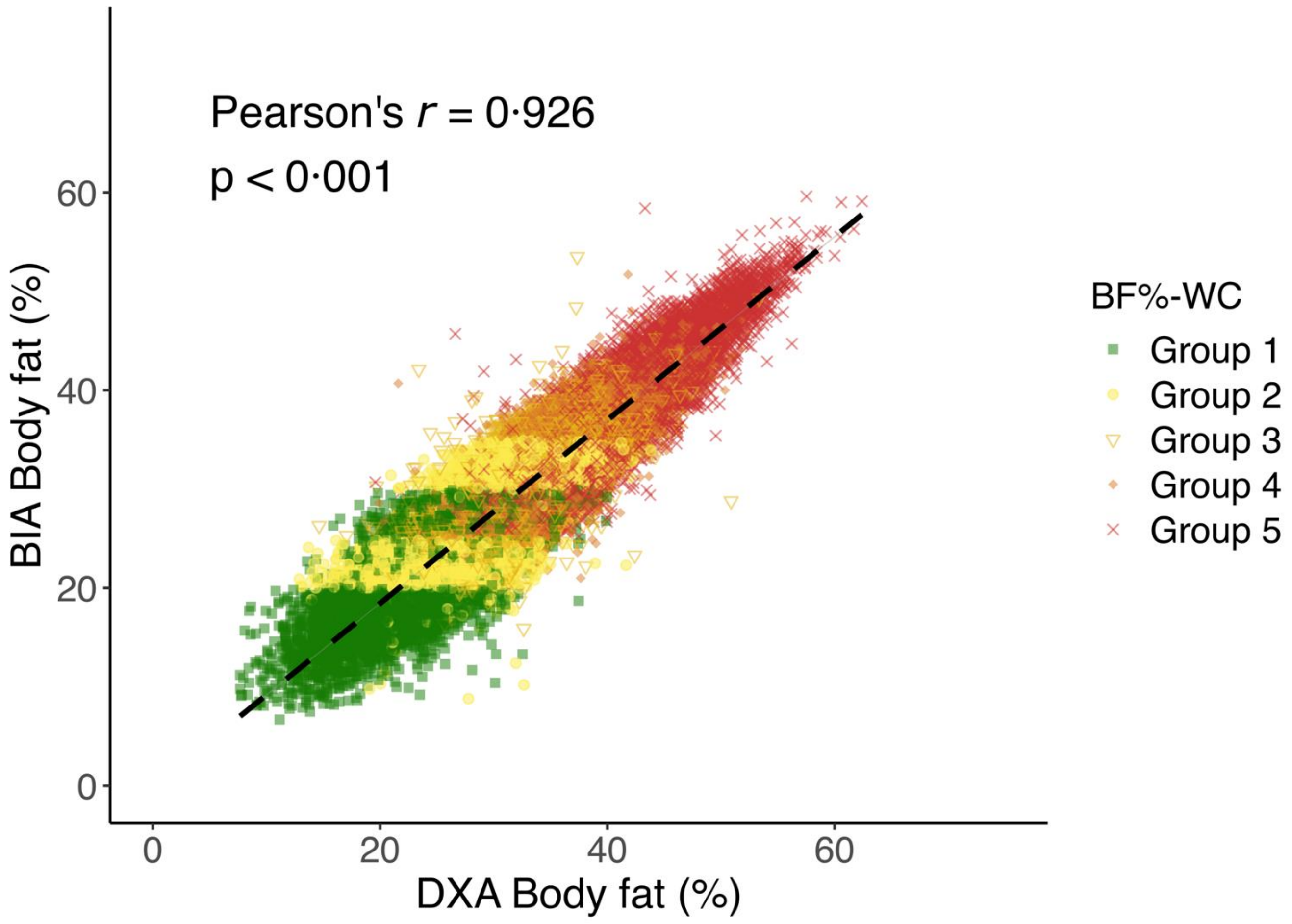

Figure S1. Correlation between DXA- and BIA-derived body fat percentage in the UK Biobank, coloured by BF%-WC groups. Pearson's correlation coefficient (*r*) and corresponding two-sided P-value are reported.

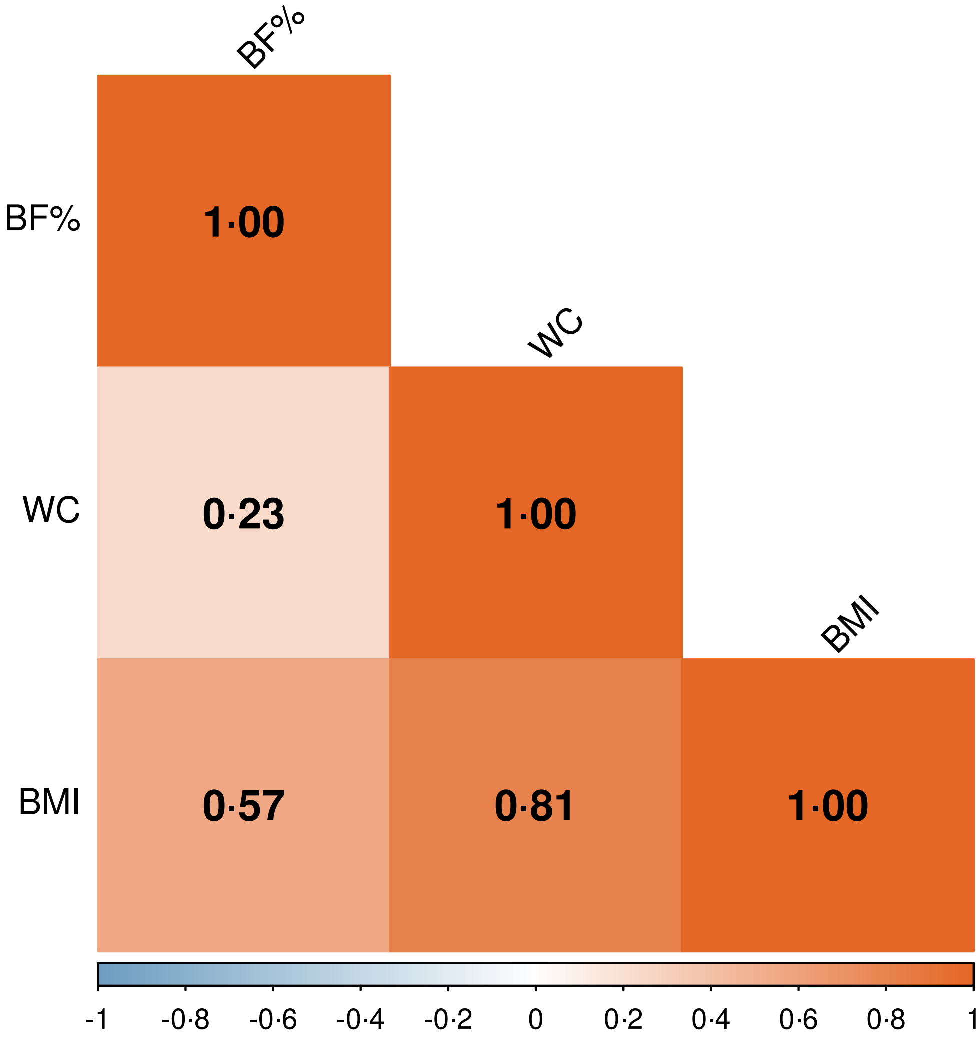

Figure S2. Correlation between BIA-derived body fat percentage (BF%), waist circumference (WC) and BMI in the UK Biobank

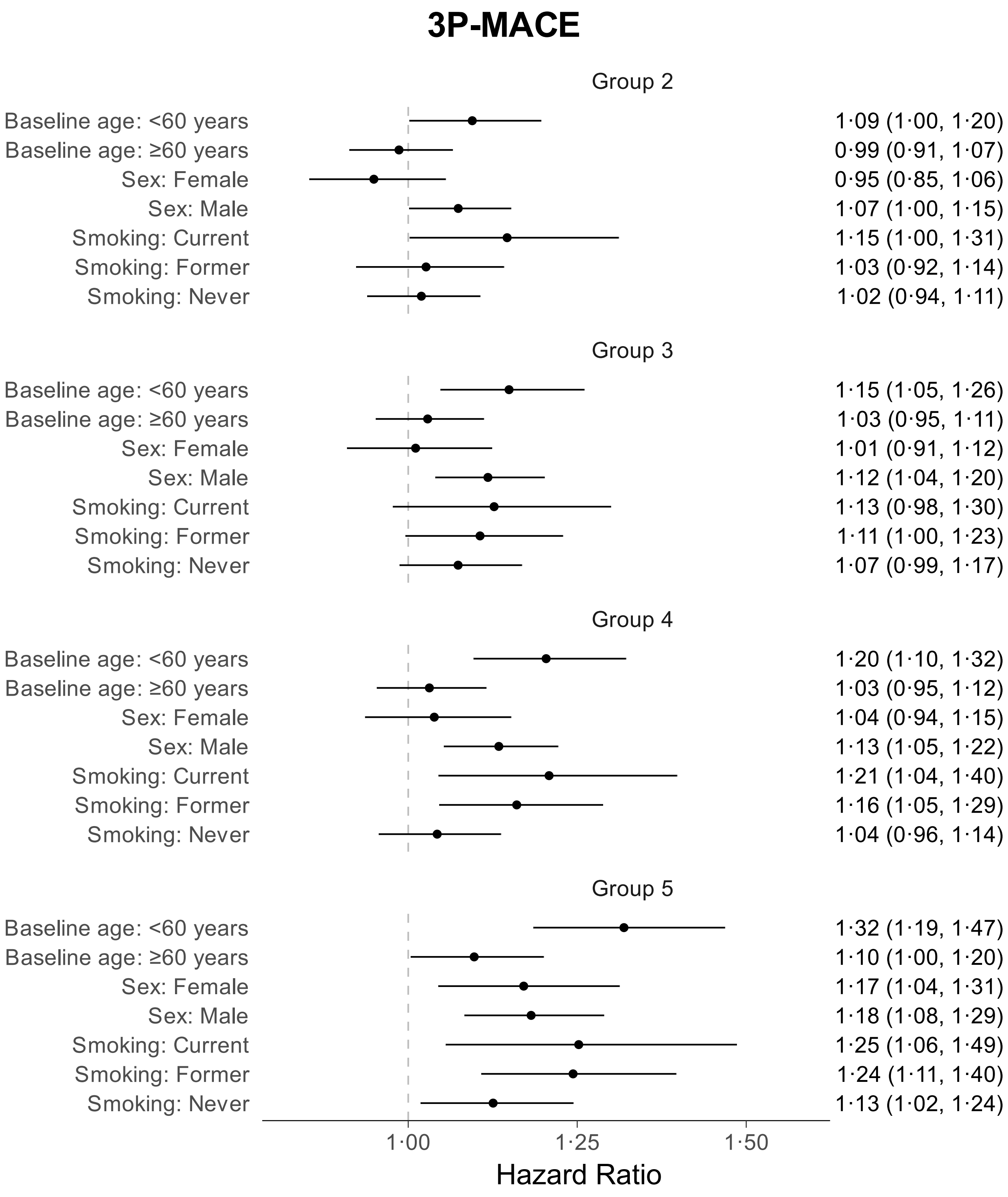

Figure S3. Subgroup analysis of BF%-WC risk groups and 3P-MACE.
Hazard ratios (HRs) with 95% confidence intervals (CIs) from Cox proportional hazards models for 3P-MACE across BF%-WC risk groups, stratified by age groups, sex, and smoking status.

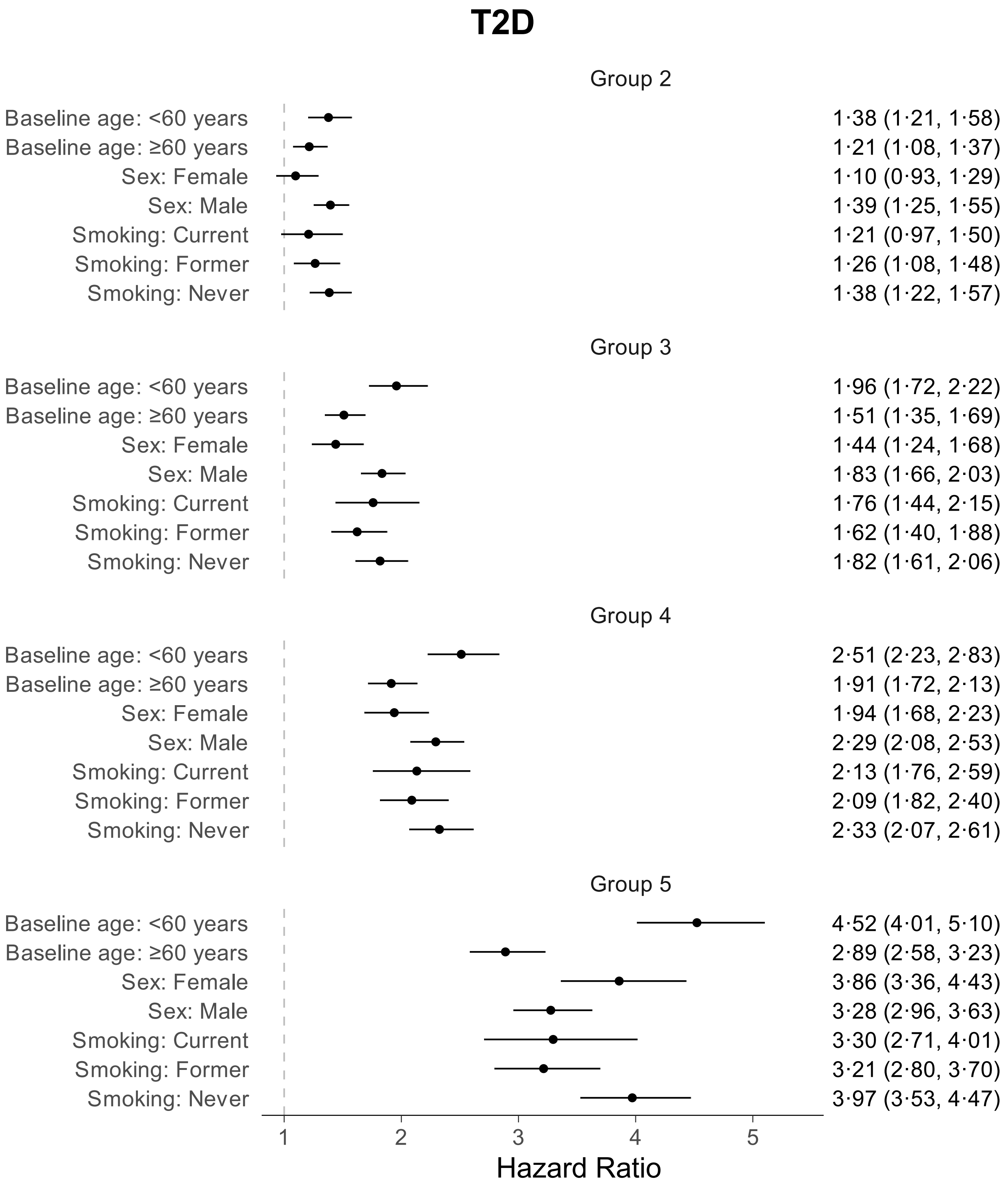

Figure S4. Subgroup analysis of BF%-WC risk groups and T2D.
Hazard ratios (HRs) with 95% confidence intervals (CIs) from Cox proportional hazards models for T2D across BF%-WC risk groups, stratified by age groups, sex, and smoking status.

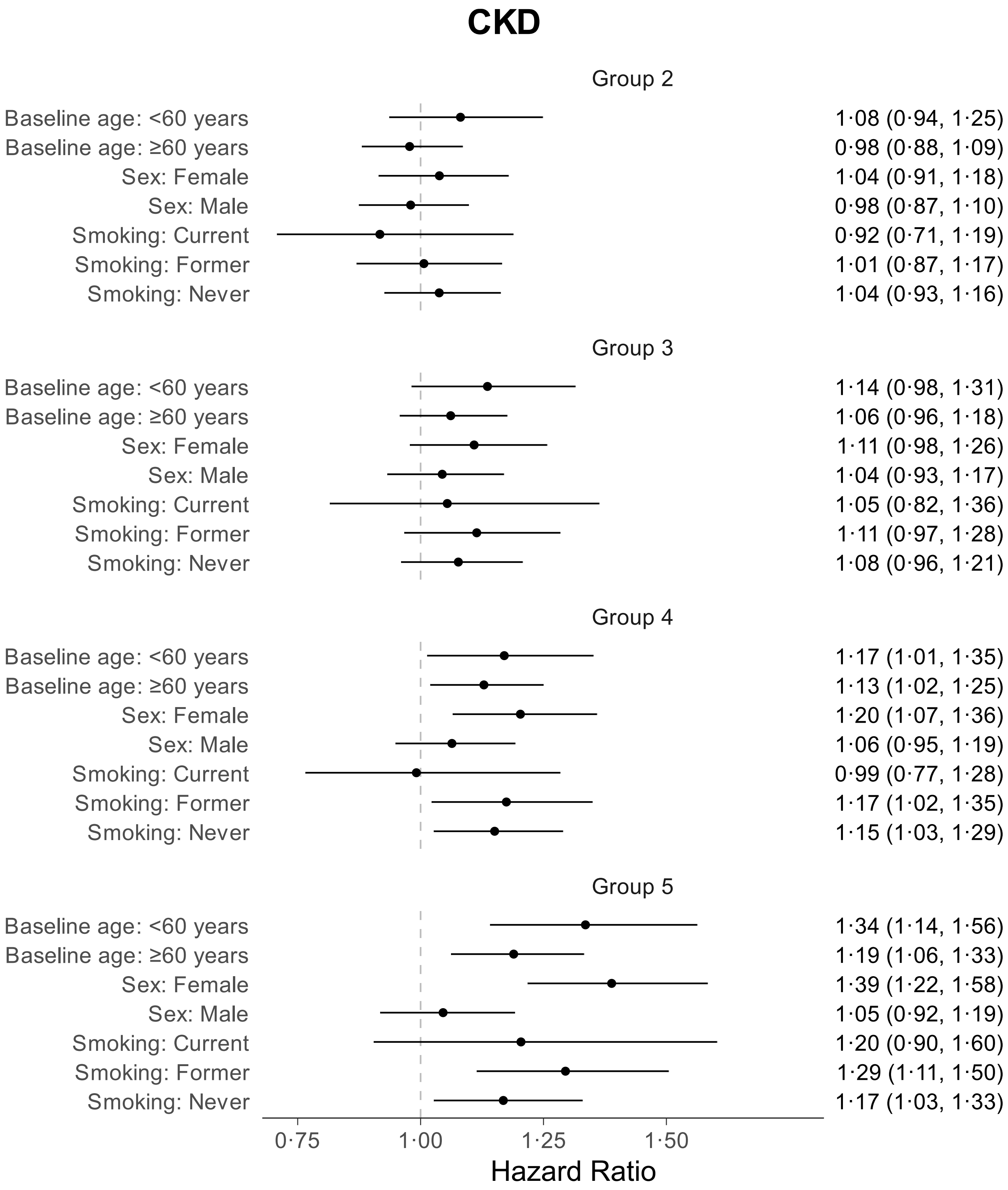

Figure S5. Subgroup analysis of BF%-WC risk groups and CKD.
Hazard ratios (HRs) with 95% confidence intervals (CIs) from Cox proportional hazards models for CKD across BF%-WC risk groups, stratified by age groups, sex, and smoking status.
